# The complete mitochondrial genome of a marine polychaete, *Ophryotrocha xiamenensis* (Annelida: Dorvilleidae)

**DOI:** 10.1080/23802359.2026.2647557

**Published:** 2026-03-26

**Authors:** Yiping Feng, Wenting Lin, Fengqi Zhang, Ruoyu Liu, Yuting Zhang, Jianming Chen, Ruanni Chen

**Affiliations:** ^a^ State Key Laboratory of Mariculture Breeding, Key Laboratory of Marine Biotechnology of Fujian Province, College of Marine Sciences, Fujian Agriculture and Forestry University, Fuzhou, China; ^b^ Fujian Key Laboratory on Conservation and Sustainable Utilization of Marine Biodiversity, Fuzhou Institute of Oceanography, College of Geography and Oceanography, Minjiang University, Fuzhou, China

**Keywords:** Mitogenome, *Ophryotrocha xiamenensis*, phylogenetic analysis, Bayesian

## Abstract

*Ophryotrocha xiamenensis* is an appropriate model for investigating regeneration and evolution. The complete mitochondrial genome is 16,111 bp in length and consists of 13 protein-coding genes (PCGs), 22 transfer RNA genes, 2 ribosomal RNA genes and a noncoding region. Phylogenetic analysis based on concatenated sequences of all 13 PCGs using the maximum-likelihood and Bayesian methods, placed *O. xiamenensis* within the ‘labronica’ clade and the order of gene arrangement was the same as that of *O. japonica*. This study contributes to the development of genetic resources and advances the understanding of phylogenetic resolution within the genus *Ophryotrocha*.

## 1. Introduction

*Ophryotrocha xiamenensis* (Chen et al. 2022) belongs to the order Eunicida, family Dorvilleidae, genus *Ophryotrocha*, and is a newly found species (Chen et al. 2022). The genus *Ophryotrocha* Claparède & Mecznikow, 1869, has been found in a wide range of habitats from shallow water to the deep sea (Alalykina and Polyakova 2022; Svensson et al. 2025). Owing to their ability to survive laboratory conditions, high fecundity, short generation times, and rapid individual growth rates, some species of *Ophryotrocha* have been used as model organisms among marine invertebrates, in the fields of genetics, reproduction, development, and regeneration (Tempestini et al. 2020; Santovito et al. 2023; Chen et al. 2024). Molecular-based taxonomic approaches have been used to identify morphologically similar species in the genus *Ophryotrocha*. Approximately 97 species in this genus, excluding *O. xiamenensis*, have been described according to GBIF data (https://www.gbif.org/); however, the complete mitochondrial genomes of only 6 species have been sequenced (Tempestini et al. 2020). The complete mitochondrial genome may serve as a foundation for thorough evolutionary investigations. The study of the genomic sequences of intermediate forms may shed light on the nuances of molecular evolution mechanisms as well as the conditions of relic species survival. In this study, we explored the complete mitochondrial genome of *O. xiamenensis* and examined its phylogenetic relationships with other species.

## 2. Materials and methods

### 2.1. Sample collections

**Figure 1.**
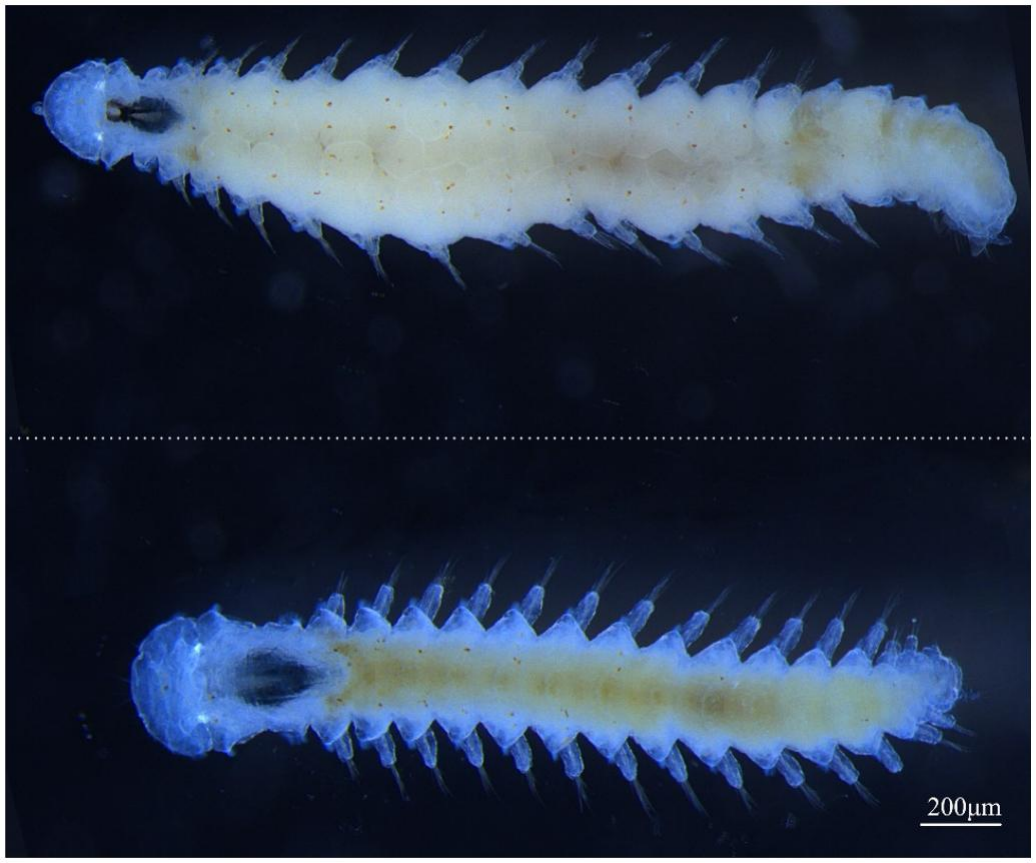
Photographs of live *O. xiamenensis*, smaller male and larger female, in dorsal view (Ruanni Chen, Minjiang University).

Specimen samples (Figure 1) were collected from Baicheng Bay, Xiamen, China (118.08E, 24.44 N), and cultured in our laboratory for more than five years. The species was identified by pairwise comparisons of *cox1* and *histone H3* sequences (Chen et al. 2022). All the samples are now deposited in the Fujian Key Laboratory on Conservation and Sustainable Utilization of Marine Biodiversity, College of Geography and Oceanography, Minjiang University under voucher number F3-7 (contact Ruanni Chen, chenruanni@mju.edu.cn).

### 2.2. Sequencing and annotation

Genomic DNA was extracted from whole worms using a TIANamp Marine Animals DNA Kit (TIANGEN, Beijing, China). Universal and specific PCR primers were designed on the basis of the *cox1* sequence from *O. xiamenensis* and conserved sequences from the genus *Ophryotrocha* using MEGA5.0 (Table 1). The fragments were amplified using Premix Taq (RR901, TaKaRa Co., Dalian, China) with initial denaturation at 95 °C for 4 min, followed by 35 cycles at 95 °C for 30 s, annealing at 45–58 °C for 1 min, and extension at 72 °C for 1 min, with a final elongation at 70 °C for 10 min after the last cycle. The amplified fragments were separated through 1.2% agarose gel electrophoresis and sequenced using the Sanger sequencing method (Figure S1, supplementary material). For assembly, the annotation of the mitogenome was performed using MITOS2 and EMBOSS Transeq (Bernt et al. 2013). Exact positions of protein-coding genes (PCGs) and rRNAs were found by searching for ORFs (employing genetic code 2, the invertebrate mitochondrion) or by homologous comparison. The circular mitochondrial genome of *O. xiamenensis* was then visualized using the Proksee web server (Grant et al. 2023).

**Table 1.** The primers used in sequencing mitochondrial genomes of *Ophryotrocha xiamenensis.*

No.	Primer	Sequence 5′-3′	Tm (°C)	Lengths
1	Ox1	CGTTTTGAAGTGGCGRRGATGT TWCCCCTTAAWGAMCCTAATAA	58	1155
2	Ox2	TAAATGTTRTCACGKAATCCKTTYC	55	1541
		CAACCATAATTAATATCMCGAGA		
3	Ox3	CAATTATTAAGGGGGTTGTTCCTC	53	1598
		CAAATCAATTCACATAACTCCTTG		
4	Ox4	ATAACTGCAGGGCATATTGT	58	1673
		TAAYWGGGTGGGGGTAGGGAAAAAG		
5	Ox5	GKGTATAGRTAYCGRATAAT	45	1581
		CCAATATCTTTATGATTWGT		
6	Ox6	AACKTSRKWTTTTTWRTTTTTA	48	1454
		GTAAAMACATCMGGRTAATCT		
8	Ox7	ATGAGCGGTGATCCCGTGTTCAG	50	1504
		GTTTCACTAGTATACTTAAAACTAG		
7	Ox8	CTATAGTAAGCTCCTTACCTTTG	55	1346
		CCAATGTGGATTGTCAAATTAT		
9	Ox9	TTAGCAGTTTTAGGGGAGTAATCT	53	1258
		TTCTAGTCCCCAATTAAGGAAC		
10	Ox10	CTGCCCGGTGCTTTTTATAGT	54	1167
		TGATATTTATCCTATGCCAAAACA		
11	Ox11	CTCTAAGTATGCGCTTTTAGGG	55	1142
		TCTTAACCACGAAAAAGTCACG		
12	Ox12	TCGTTTAAGAGCCTTCTATAGTT	51	870
		ATTCGGTATAAGGTACTCACTCA		
13	Ox13	GGCATTAATTATAACAACACTATG	49	1393
		GGAAAACCGTTAATTGTAGTATAG		

### 2.3. Phylogenetic analysis

Molecular phylogenetic analyses were performed with datasets from all 13 PCGs through MEGA 5.05 software (Tamura et al. 2011). Each PCG was separately aligned, after which the concatenated and poorly aligned regions were removed. In total, 11 terminal taxa were used in the analyses—7 species from the genus *Ophryotrocha*, including *O. xiamenensis*, and 3 species from the other families of Polychaeta; the tree was rooted using *Sipunculus nudu*. The aligned sequences were used as datasets to generate the genetic distance using Kimura’s two-parameter (K2P) model. On the basis of the K2P distances (Table S1), we calculated the interspecific genetic differences among the closest taxa. The phylogenetic trees were constructed by the maximum likelihood method (ML) using MEGA software, with 1,000 bootstrap pseudo replicates. ModelFinder was used to select the best-fit model for Bayesian analysis by using PhyloSuite version 1.2.3 (Zhang et al. 2020). Bayesian inference (BI) phylogenies were inferred under the TVM+I + G + F model (2 parallel runs, 200000 generations), in which the initial 25% of the sampled data were discarded as burn-in. FigTree v1.4.4 was used to visualize the tree. There are neither trans-splicing nor cis-splicing genes in the mitochondrial genome.

## 3. Results

**Figure 2.**
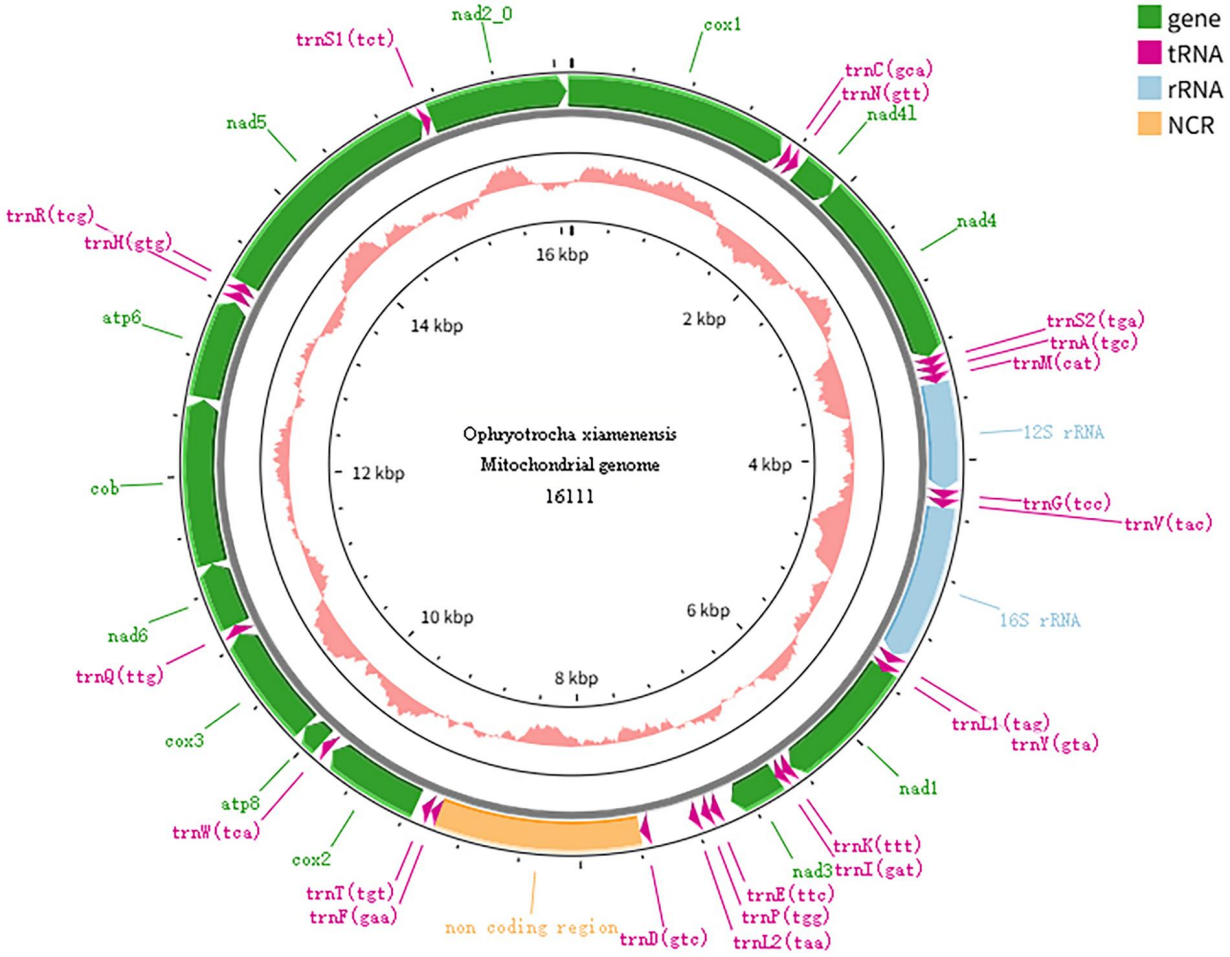
Circular map of the complete mitochondrial genome of *ophryotrocha xiamenensis*. The complete mitochondrial genome was 16,111 bp in length. Genes were shown with standard abbreviations. The outer circle indicated the plus strand (outer line) and the minus strand (inner line), while all genes were coded on plus strand. The inner pink bars indicated the GC content, and the Middle line represented 50%.

We assembled a complete mitochondrial genome of *O. xiamenensis* which has been maintained in our laboratory for more than five years. The complete mitogenome (GenBank Accession No. PV831793) is 16,111 bp long and A + T biased. Sanger sequencing chromatograms of the *O. xiamenensis* mitogenome are provided in the supplementary material. Thirty-seven genes were identified from the mitogenome sequence, including 13 PCGs, 2 ribosomal genes (12S and 16S), 22 transport RNA genes, and 1 noncoding region (NCR). The NCR of the mitochondrial genome was located between *trnD* and *trnF*. The total base composition was A (26.8%), T (37.8%), G (24.1%), and C (11.3%). All the genes were coded on the plus strand (Figure 2), and no introns were found (see Figure S2, supplementary material). The total length of the 13 PCGs and NCR was 10,994 bp and 6 overlapping regions were present in the whole mitochondrial genome, including 1 to 17 bp (see Table S2, supplementary material). Most of the genes started with an ATG codon and ended with a TAA/TAG stop codon. Specifically, for *cox1* and *cytb,* ATT and GTG as start codons.

**Figure 3.**
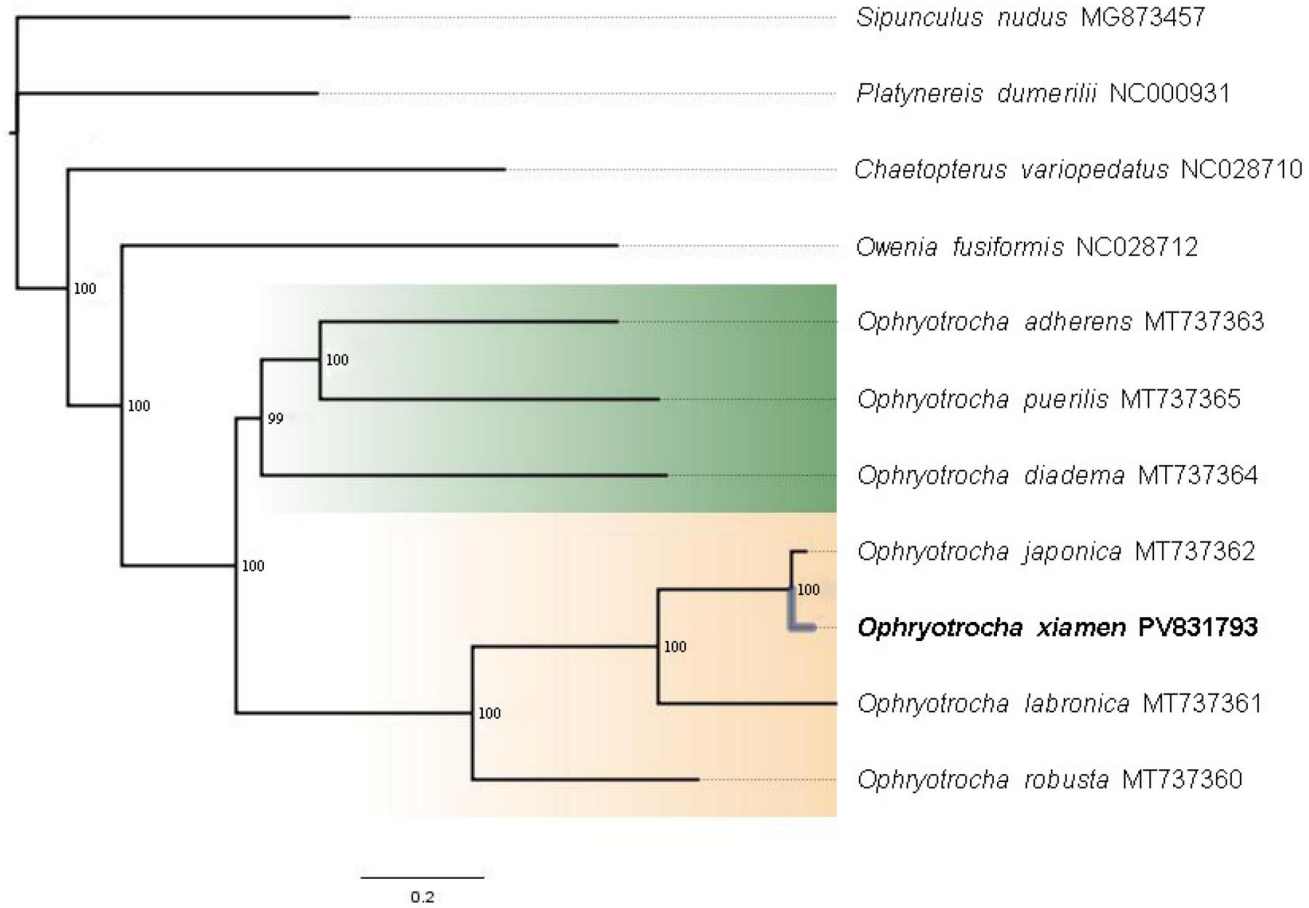
Phylogenetic tree was constructed using 13 protein-coding genes of the complete mitochondrial genome through PhyloSuite v1.2.3. Bayesian posterior probabilities were shown at each node. The following sequences were used: *Sipunculus nudus* MG873457 (Zhong et al. 2018), *Platynereis dumerilii* NC000931 (Won et al. 2013), *Chaetopterus variopedatus* NC028710 (Weigert et al. 2016), *Owenia fusiformis* NC028712 (Weigert et al. 2016), *Ophryotrocha adherens* MT737363 (Tempestini et al. 2020), *Ophryotrocha puerilis* MT737365 (Tempestini et al. 2020), *Ophryotrocha diadema* MT737364 (Tempestini et al. 2020), *Ophryotrocha japonica* MT737362 (Tempestini et al. 2020), *Ophryotrocha xiamenensis* PV831793, *Ophryotrocha labronica* MT737361(Tempestini et al. 2020), and *Ophryotrocha robusta* MT737360 (Tempestini et al. 2020).

After the poorly aligned positions were removed, a total of 12550 bp of 13 PCGs were used for phylogenetic analyses. Phylogenetic analyses revealed similar tree topologies regardless of whether the ML or Bayesian approach was used; therefore, only the results from the Bayesian analysis are shown (Figure 3). Maximum-likelihood and BI revealed phylogenetic relationships within the genus *Ophryotrocha*, indicating that *O. xiamenensis* fell within the ‘labronica’ clade. *O. xiamenensis* presented the same gene order as *O. japonica* did, except for *trnD*. The 13 PCGs of *O. xiamenensis* and *O. japonica* were in the same order. The positions of *trnL2*, *trnD*, and *trnP* could help to identify related species in the ‘labronica’ clade. The mitochondrial genome, especially its gene arrangement, is valuable for studying evolutionary relationships among the genus *Ophryotrocha*.

## 4. Discussion and conclusion

The genus *Ophryotrocha* has been used as a model organism in several fields of comparative and evolutionary biology of marine invertebrates. Herein, the complete mitogenome of *O. xiamenensis* was sequenced and found to be 16,111 bp in length, including 13 PCGs, 22 tRNAs, 2 rRNAs and 1 NCR. Notably, *cox1* and *cytb* used ATT and GTG were used as start codons for *cox1* and *cytb*, which were also found in *O. japonica* and *O. labornica* (Tempestini et al. 2020). Previous studies have demonstrated that mitogenomes within the genus *Ophryotrocha* exhibit high dynamism in terms of gene order (Tempestini et al. 2020; Struck et al. 2023). In this study, the gene order of *O. xiamenensis* was most similar to that of *O. japonica* but differed from that of *O. labronica*, indicating that gene arrangement varies between species (Table S3).

The phylogenetic tree based on all available genomes of species within the genus *Ophryotrocha* has improved our understanding of their evolutionary process. Only the position of *O. diadema* changes between the two mitochondrial trees on the basis of 13 PCGs or *cox1*/Histone H3 sequences (Dahlgren et al. 2001; Tempestini et al. 2020; Pruitt 2021). *O. xiamenensis*, *O. japonica* and *O. labronica*, have the same gene order, and differ from other annelids, such as *O. adherens, O. diadema, O. robusta,* and *Capitella teleta* (Tempestini et al. 2020; Tilic and Rouse 2024; Su et al. 2025). Overall, our findings contribute to the exploration of the evolution, high biodiversity, and phylogenetic relationships among annelid taxa.

## Supplementary Material

Initial The complete mitochondrial genome of a marine polychaete.docx

Revised 1113 The complete mitochondrial genome of a marine polychaete.docx

Editing Certificate.pdf

Supplementary material.pdf

clean copy The complete mitochondrial genome of a marine polychaete.docx

## Data Availability

The genome sequence data that support the findings of this study are openly available in GenBank of NCBI at https://www.ncbi.nlm.nih.gov/ under the accession no. PV831793. The chromatographic raw data used to generate the results are available at Zenodo: https://doi.org/10.5281/zenodo.17473716.

## References

[CIT0001] Alalykina IL, Polyakova NE. 2022. New species of *Ophryotrocha* (Annelida: dorvilleidae) associated with deep-sea reducing habitats in the Bering Sea, Northwest Pacific. Deep Sea Res Part II. 206:105217. 10.1016/j.dsr2.2022.105217

[CIT0002] Bernt M et al. 2013. MITOS: improved de novo metazoan mitochondrial genome annotation. Mol Phylogenet Evol. 69(2):313–319. 10.1016/j.ympev.2012.08.023 22982435

[CIT0003] Chen R, Cheng Y, Zhang Y, Chen J. 2024. Identification and expression analysis of Oxfibrillin gene involved in the regeneration process of *Ophryotrocha xiamen* (Annelida, Dorcilleidae). Dev Comp Immunol. 151:105102. 10.1016/j.dci.2023.105102 37995918

[CIT0004] Chen R, Mukhtar I, Wei S, Wu S, Chen J. 2022. Morphological and molecular features of early regeneration in the marine annelid *Ophryotrocha xiamen*. Sci Rep. 12(1):1799. 10.1038/s41598-022-04870-3 35110576 PMC8810878

[CIT0005] Dahlgren TG, Akesson B, Schander C, Halanych KM, Sundberg P. 2001. Molecular phylogeny of the model annelid *Ophryotrocha*. Biol Bull. 201(2):193–203. 10.2307/1543334 11687391

[CIT0006] Grant JR et al. 2023. Proksee: in-depth characterization and visualization of bacterial genomes. Nucleic Acids Res. 51(W1):W484–W492. 10.1093/nar/gkad326 37140037 PMC10320063

[CIT0007] Pruitt J. 2021. Phylogeny of *Ophryotrocha* (Annelida Dorvilleidae) Revisited, with Description of Six New Species from Eastern Pacific Seeps and Whalefalls [Master’s thesis]. University of California San Diego.

[CIT0008] Santovito A, Pappalardo A, Nota A, Prearo M, Schleicherová D. 2023. Lymnaea stagnalis and *Ophryotrocha diadema* as model organisms for studying genotoxicological and physiological effects of benzophenone-3. Toxics. 11(10):827. 10.3390/toxics11100827 37888678 PMC10610920

[CIT0009] Su X et al. 2025. Substantial mitochondrial gene order rearrangements and differential evolution rates within the family Capitellidae (Annelida). ZSE. 101(3):955–967. 10.3897/zse.101.144081

[CIT0010] Struck TH, Golombek A, Hoesel C, Dimitrov D, Elgetany AH. 2023. Mitochondrial genome evolution in Annelida—a systematic study on conservative and variable gene orders and the factors influencing its evolution. Syst Biol. 72(4):925–945. 10.1093/sysbio/syad023 37083277 PMC10405356

[CIT0011] Svensson SGB, Meier S, Mjøs SA, Strohmeier T, Jansen HM. 2025. *Ophryotrocha craigsmithi* (Wiklund, Glower & Dahlgren, 2009) has a high capacity to modify lipids from aquaculture waste and synthesize long-chain polyunsaturated fatty acids. Aquaculture. 608:742746. 10.1016/j.aquaculture.2025.742746

[CIT0012] Tamura K et al. 2011. MEGA5: molecular evolutionary genetics analysis using maximum likelihood, evolutionary distance, and maximum parsimony methods. Mol Biol Evol. 28(10):2731–2739. 10.1093/molbev/msr121 21546353 PMC3203626

[CIT0013] Tempestini A et al. 2020. Extensive gene rearrangements in the mitogenomes of congeneric annelid species and insights on the evolutionary history of the genus *Ophryotrocha*. BMC Genomics. 21(1):815. 10.1186/s12864-020-07176-8 33225885 PMC7682095

[CIT0014] Tilic E, Rouse GW. 2024. Hardly Venus’s servant-morphological adaptations of Veneriserva to an endoparasitic lifestyle and its phylogenetic position within Dorvilleidae (Annelida). Org Divers Evol. 24(1):67–83. 10.1007/s13127-023-00633-8

[CIT0015] Weigert A et al. 2016. Evolution of mitochondrial gene order in Annelida. Mol Phylogenet Evol. 94(Pt A):196–206. 10.1016/j.ympev.2015.08.008 26299879

[CIT0016] Won EJ, Rhee JS, Shin KH, Lee JS. 2013. Complete mitochondrial genome of the marine polychaete, *Perinereis nuntia* (Polychaeta, Nereididae). Mitochondrial DNA. 24(4):342–343. 10.3109/19401736.2012.760082 23379328

[CIT0017] Zhang D et al. 2020. PhyloSuite: an integrated and scalable desktop platform for streamlined molecular sequence data management and evolutionary phylogenetics studies. Mol Ecol Resour. 20(1):348–355. 10.1111/1755-0998.13096 31599058

[CIT0018] Zhong S, Zhao Y, Zhang Q, Chen X. 2018. The complete mitochondrial genome of the cryptic species in peanut worm *Sipunculus nudus* (Sipuncula, Sipunculidae) from Beibu Bay. Mitochondrial DNA B Resour. 3(2):484–485. 10.1080/23802359.2018.1463830 33474213 PMC7800096

