# Supplementary material for: The complete mitochondrial genome of a marine polychaete, Ophryotrocha xiamenensis (Annelida: Dorvilleidae)
Source: Mitochondrial DNA B Resour. 2026 Mar 26;11(5):567–71. doi: 10.1080/23802359.2026.2647557 (PMC13022994; doi:10.1080/23802359.2026.2647557)
Supplement: Revised 1113 The complete mitochondrial genome of a marine polychaete.docx [file TMDN_A_2647557_SM7437.docx]

**The complete mitochondrial genome of a marine polychaete, *Ophryotrocha xiamenensis* (Annelida: Dorvilleidae)**

Yiping Feng^1, 2^, Wenting Lin^2^, Fengqi Zhang^2^, Ruoyu Liu^1^, Yuting Zhang^2^, Jianming Chen*^2^, Ruanni Chen*^2^

1 State Key Laboratory of Mariculture Breeding, Key Laboratory of Marine Biotechnology of Fujian Province, College of Marine Sciences, Fujian Agriculture and Forestry University, Fuzhou 350002, China

2 Fujian Key Laboratory on Conservation and Sustainable Utilization of Marine Biodiversity, Fuzhou Institute of Oceanography, College of Geography and Oceanography, Minjiang University, Fuzhou, 350108, China

**Correspondence:**

(1) Ruanni Chen, chenruanni@163.com; (2) Jianming Chen, chenjm@mju.edu.cn.

**Abstract**

*Ophryotrocha xiamenensis*, a small opportunistic worm, has been found to be an appropriate model for investigating regeneration and evolution. In this study, the complete mitochondrial genome of *O. xiamenensis* was sequenced by Sanger platform. It was 16,111bp in length and consisted of 13 protein coding genes (PCGs), 22 transfer RNA genes, 2 ribosomal RNA genes and a non-coding region. Phylogenetic analysis based on concatenated sequences of all 13 PCGs using the maximum-likelihood and Bayesian methods placed *O. xiamenensis* within the ‘labronica’ clade and the order of gene arrangement was same with *O. japonica*. This study would enrich genetic resources and advance the understanding of phylogenetic resolution within the genus *Ophryotrocha*.

**Keywords**

Mitogenome, *Ophryotrocha* *xiamenensis*, phylogenetic analysis, Bayesian

**1. Introduction**

*Ophryotrocha xiamenensis* Chen, 2022, belonged to the order Eunicida, family Dorvilleidae, genus *Ophryotrocha*, is a newly found species (Chen *et al.*, 2022). The genus *Ophryotrocha* Claparède & Mecznikow, 1869 has been found in a wide range of habitats from shallow water to the deep sea (Alalykina and Polyakova, 2022; Svensson *et al.*, 2025). Due to their capability of laboratory maintenance, high fecundity, short generation time, and rapid individual growth rate, some species of *Ophryotrocha* have been used as model organisms of marine invertebrates in the fields of genetics, reproduction, development, and regeneration (Tempestini *et al.*, 2020; Santovito *et al.*, 2023; Chen *et al.*, 2024). Molecular based taxonomic approaches have been used to identify morphologically similar species in genus *Ophryotrocha*. Approximately 97 species in this genus, excluding *O. xiamenensis*, have been described according to the GBIF data (https://www.gbif.org/), however, the complete mitochondrial genomes of only 6 species have been sequenced (Tempestini *et al.*, 2020). The complete mitochondrial genome may serve as a foundation for thorough evolutionary investigations. The study of genomic sequences of intermediate forms may shed light on nuances of molecular evolution mechanisms as well as conditions of relic species survival. In this study, we explored the complete mitochondrial genome of *O. xiamenensis* and examine its phylogenetic relationship with others.

**2. Material and methods**

**2.1 Sample collections**

Specimen samples (Figure 1) were collected from Baicheng Bay, Xiamen, China (118.08E, 24.44 N), and cultured in our laboratory for more than five years. The species was identified by pairwise comparisons of COI and histone H3 sequences(Chen *et al.*, 2022). All specimens are now deposited in the Fujian Key Laboratory on Conservation and Sustainable Utilization of Marine Biodiversity, College of Geography and Oceanography, Minjiang University under the voucher number F3-7 (contact Ruanni Chen, chenruanni@mju.edu.cn).


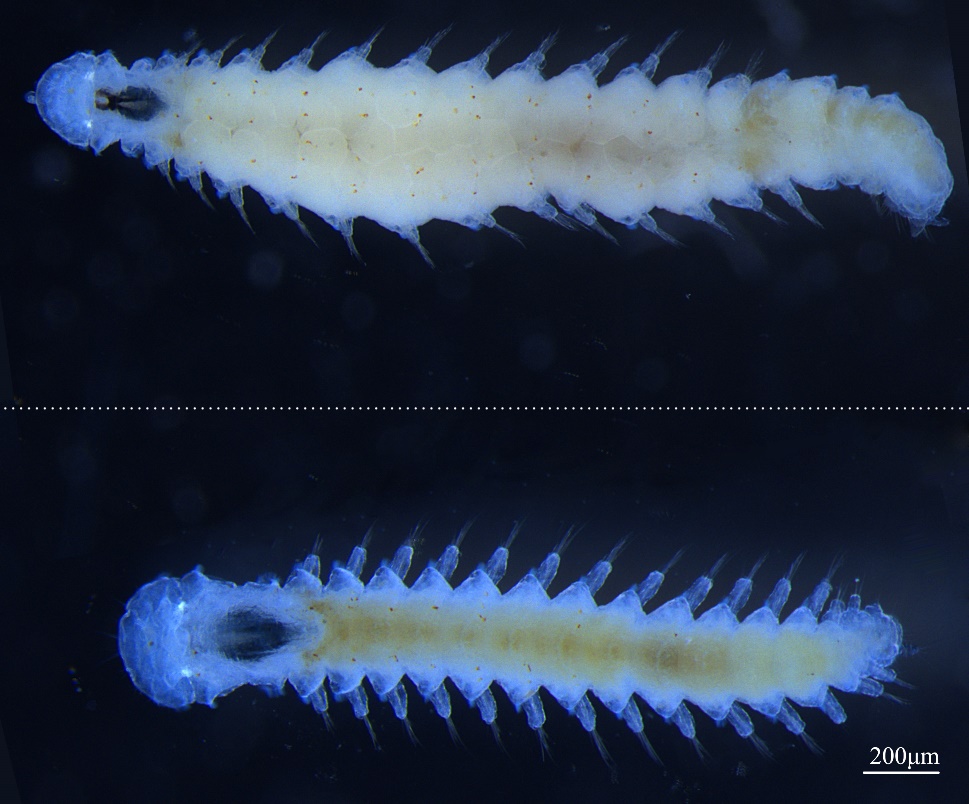


Figure 1. Photographs of live *O. xiamenensis*, smaller male and larger female, in dorsal view (Ruanni Chen, Minjiang University).

**2.2 Sequencing and annotation**

Genomic DNA was extracted form whole worms using TIANamp Marine Animals DNA Kit (TIANGEN, Beijing, China). The universal and specific PCR primers were designed based on *cox1* sequence from *O. xiamenensis* and conserved sequences from genus *Ophryotrocha* using MEGA5.0 (Table 1). The fragments were amplified using Premix Taq (RR901, TaKaRa Co., Dalian, China) with an initial denaturation at 95 °C for 4 min, followed by 35 cycles at 95 °C for 30 s, annealing at 45–58 °C for 1 min, and extension at 72 °C for 1 min, with a final elongation at 70 °C for 10 min after the last cycle. The amplification fragments were separated by 1.2% agarose gel electrophoresis and sequenced using Sanger sequencing method (see Figure S1, supplementary material). For assembly, the annotation of the mitogenome was made with the MITOS2 and EMBOSS Transeq (Bernt *et al.*, 2013). Exact positions of protein-coding genes (PCGs) and rRNAs were found by searching for ORFs (employing genetic code 2, the invertebrate mitochondrion) or homologic comparison. The circular mitochondrial genome of *O. xiamenensis* was then visualized using Proksee web server (Grant *et al.*, 2023).

Table 1. The primers used in sequencing mitochondrial genomes of *Ophryotrocha xiamenensis*.

| No. | Primer | Sequence 5’-3’ | Tm (℃) | Lengths |
| --- | --- | --- | --- | --- |
| 1 | Ox1 | CGTTTTGAAGTGGCGRRGATGT  TWCCCCTTAAWGAMCCTAATAA | 58 | 1155 |
| 2 | Ox2 | TAAATGTTRTCACGKAATCCKTTYC | 55 | 1541 |
|  |  | CAACCATAATTAATATCMCGAGA |  |  |
| 3 | Ox3 | CAATTATTAAGGGGGTTGTTCCTC | 53 | 1598 |
|  |  | CAAATCAATTCACATAACTCCTTG |  |  |
| 4 | Ox4 | ATAACTGCAGGGCATATTGT | 58 | 1673 |
|  |  | TAAYWGGGTGGGGGTAGGGAAAAAG |  |  |
| 5 | Ox5 | GKGTATAGRTAYCGRATAAT | 45 | 1581 |
|  |  | CCAATATCTTTATGATTWGT |  |  |
| 6 | Ox6 | AACKTSRKWTTTTTWRTTTTTA | 48 | 1454 |
|  |  | GTAAAMACATCMGGRTAATCT |  |  |
| 8 | Ox7 | ATGAGCGGTGATCCCGTGTTCAG | 50 | 1504 |
|  |  | GTTTCACTAGTATACTTAAAACTAG |  |  |
| 7 | Ox8 | CTATAGTAAGCTCCTTACCTTTG | 55 | 1346 |
|  |  | CCAATGTGGATTGTCAAATTAT |  |  |
| 9 | Ox9 | TTAGCAGTTTTAGGGGAGTAATCT | 53 | 1258 |
|  |  | TTCTAGTCCCCAATTAAGGAAC |  |  |
| 10 | Ox10 | CTGCCCGGTGCTTTTTATAGT | 54 | 1167 |
|  |  | TGATATTTATCCTATGCCAAAACA |  |  |
| 11 | Ox11 | CTCTAAGTATGCGCTTTTAGGG | 55 | 1142 |
|  |  | TCTTAACCACGAAAAAGTCACG |  |  |
| 12 | Ox12 | TCGTTTAAGAGCCTTCTATAGTT | 51 | 870 |
|  |  | ATTCGGTATAAGGTACTCACTCA |  |  |
| 13 | Ox13 | GGCATTAATTATAACAACACTATG | 49 | 1393 |
|  |  | GGAAAACCGTTAATTGTAGTATAG |  |  |

**2.3 Phylogenetic analysis**

Molecular phylogenetic analyses were performed with data sets from all 13 PCGs through MEGA 5.05 software (Tamura *et al.*, 2011). Each PCG was separately aligned and then concatenated and poorly aligned regions were removed. In total, 11 terminal taxa were used in the analyses, 7 species from the genus *Ophryotrocha* including *O. xiamenensis*, 3 species from the other families of Polychaeta, and rooted using *Sipunculus nudu*. The aligned sequences were used as data sets to generate the genetic distance using Kimura’s two-parameter (K2P) model. Based on the K2P distances (Table S1), we calculated the interspecific genetic differences among the closest taxa. The phylogenetic trees were constructed by the maximum likelihood method (ML) using MEGA software, with 1,000 bootstrap pseudo replicates. ModelFinder was used to select the best-fit model in Bayesian analysis by using PhyloSuite version1.2.3(Zhang *et al.*, 2020). Bayesian Inference (BI) phylogenies were inferred under TVM+I+G+F model (2 parallel runs, 200000 generations), in which the initial 25% of sampled data were discarded as burn-in. FigTree v1.4.4 was used to visualize the tree. There are neither trans-splicing nor cis-splicing genes in the mitochondrial genome.

**3. Results**

We assembled a complete mitochondrial genome of the *Ophryotrocha xiamenensis* which were maintained in our laboratory for more than five years. The complete mitogenome (GenBank Accession No. PV831793) is 16,111 bp long and A + T biased. The Sanger sequencing chromatograms of *O. xiamenensis* mitogenome were provide in Supplementary material. Thirty-seven genes of the mitogenome sequence were identified, including 13 protein-coding genes (PCGs), 2 ribosomal genes (12S and 16S), 22 transport RNA genes, and 1 non-coding region (NCR). The NCR of the mitochondrial genome existed between the *trnD* and *trnF*. The total base composition was A (26.8%), T (37.8%), G (24.1%), and C (11.3%). All genes were coded on the plus strand (Figure 2) and no intron was found (see Figure S2, supplementary material). The total length of 13 PCGs and NCR was 10,994 bp and 6 overlapping regions presented in the whole mitochondrial genome with 1 to 17 bp (see Table S2, supplementary material). Most of the genes started with the ATG codon and finished with the TAA/TAG stop codon. Specially, the *cox1* and *cytb* used ATT and GTG as start codons.


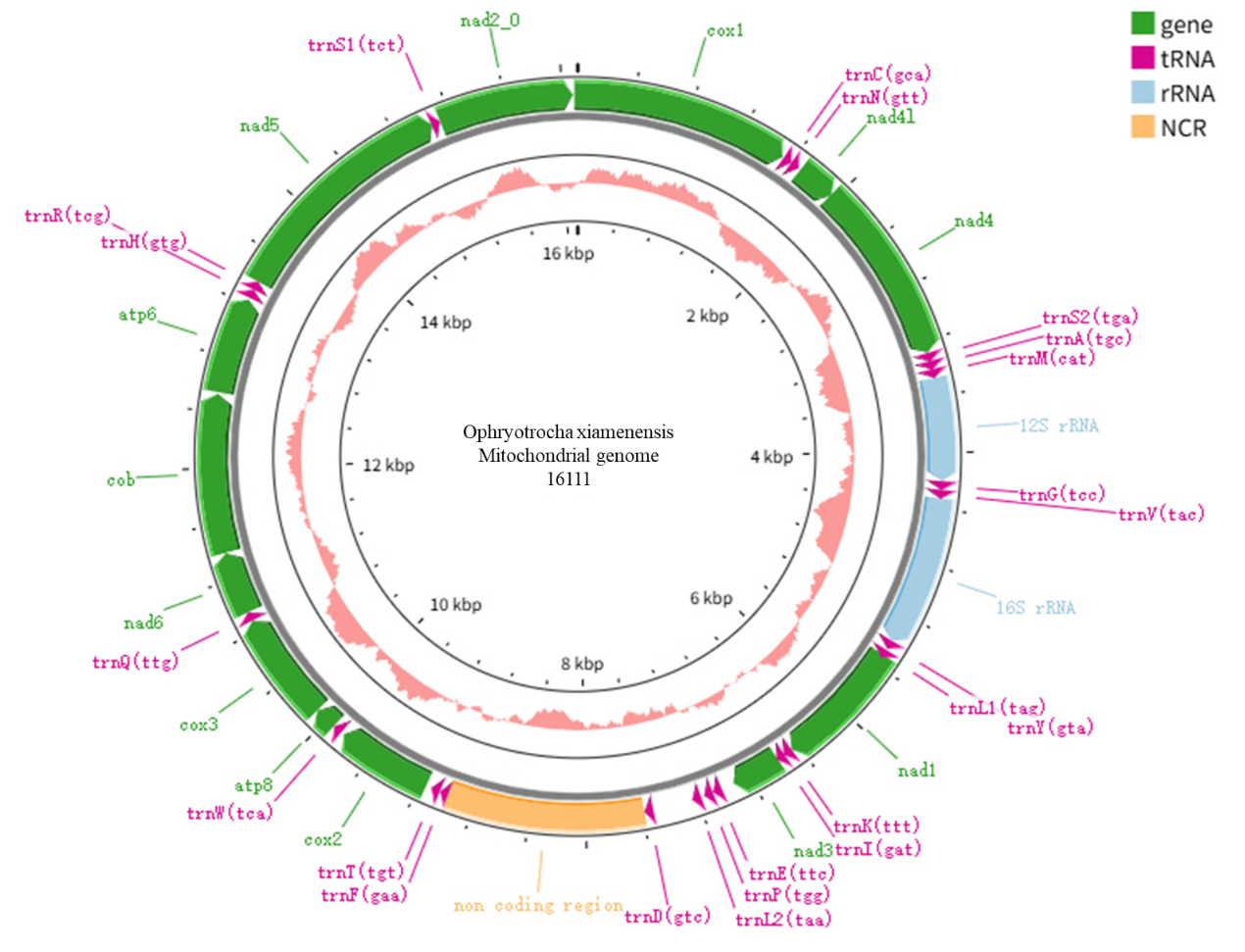


Figure 2. Circular map of the complete mitochondrial genome of *Ophryotrocha xiamenensis*. The complete mitochondrial genome was 16,111 bp in length. Genes were shown with standard abbreviations. The outer circle indicated the plus strand (outer line) and the minus strand (inner line), while all genes were coded on plus strand. The inner pink bars indicated the GC content, and the middle line represented 50%.

After removing the poorly aligned positions, a total of 12550 bp of 13 PCGs were used for phylogenetic analyses. Phylogenetic analyses resulted in similar tree topologies regardless of ML or Bayesian approaches were used, therefore, only the results from the Bayesian analysis were shown (Figure 3). Maximum-likelihood and BI revealed the phylogenetic relationships within the genus *Ophryotrocha*, indicating that *O. xiamenensis* fell within the ‘labronica’ clade. *O. xiamenensis* showed the same gene order with *O. japonica*, except *trnD*. For 13 PCGs, *O. xiamenensis* and *O. japonica* were in the same order. The position of *trnL2*, *trnD*, *trnP* could help to identified related species in ‘labronica’ clade. The mitochondrial genome, especially gene arrangement, will be valuable for studying evolutionary relationships among the genus *Ophryotrocha*.


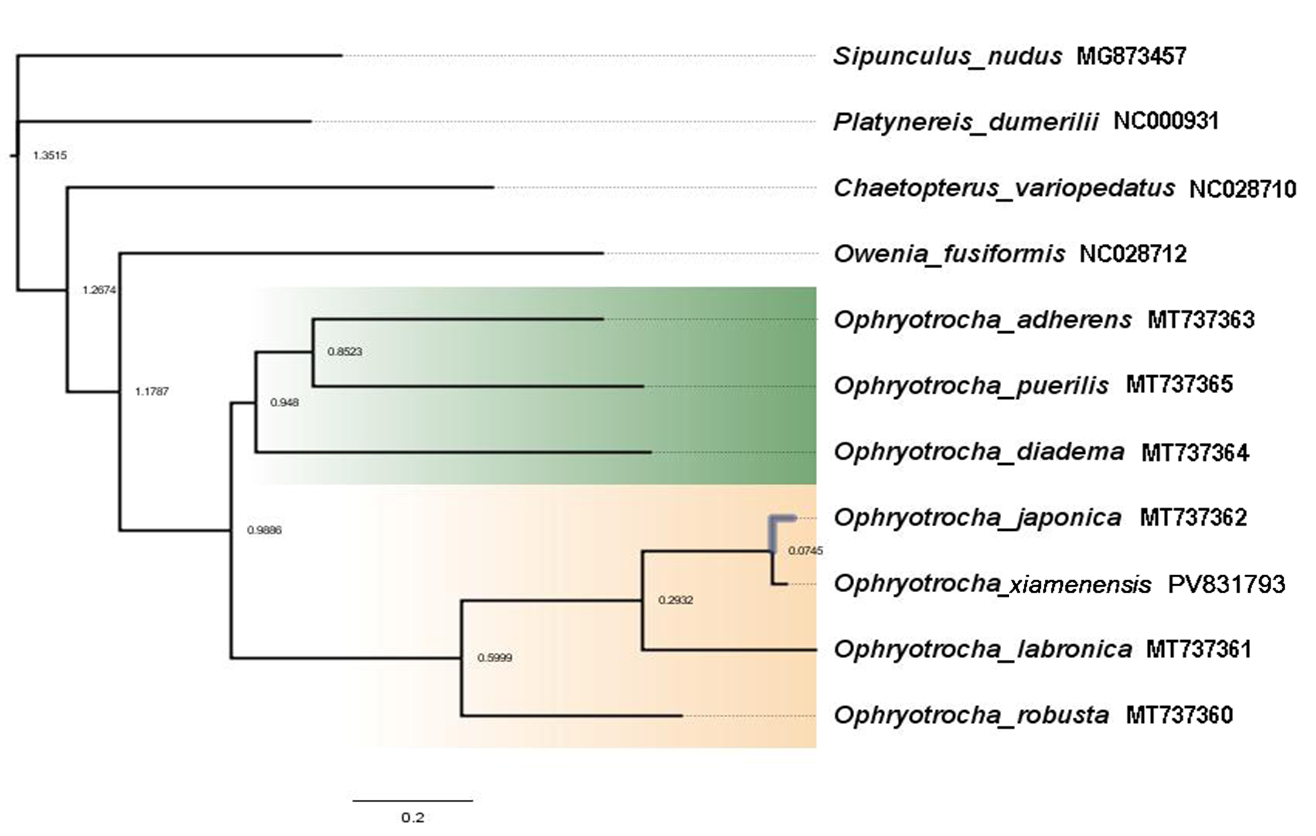


Figure 3. Phylogenetic tree was constructed using 13 protein-coding genes of the complete mitochondrial genome through PhyloSuite v1.2.3. The following sequences were used: *Sipunculus nudus* MG873457 (Zhong *et al*., 2018), *Platynereis dumerilii* NC000931 (Won *et al.*, 2013), *Chaetopterus variopedatus* NC028710 (Weigert *et al*.,2015), *Owenia fusiformis* NC028712 (Weigert *et al*.,2015), *Ophryotrocha adherens* MT737363 (Tempestini *et al*.,2020), *Ophryotrocha puerilis* MT737365 (Tempestini *et al*.,2020), *Ophryotrocha diadema* MT737364 (Tempestini *et al*.,2020), *Ophryotrocha japonica* MT737362 (Tempestini *et al*.,2020), *Ophryotrocha xiamenensis* PV831793, *Ophryotrocha labronica* MT737361(Tempestini *et al*.,2020), and *Ophryotrocha robusta* MT737360 (Tempestini *et al*.,2020).

**4. Discussion and Conclusion**

The genus *Ophryotrocha* has been used as a model organism in several fields of comparative and evolutionary biology of marine invertebrates. Herein, the complete mitogenome of *O. xiamenensis* was sequenced and found to be 16,111 bp in length, 13 PCGs, 22 tRNAs, 2 rRNAs and 1 NCR. Notably, *cox1* and *cytb* used ATT and GTG as start codons, which was also found in *O. japonica* and *O. labornica* (Tempestini *et al.*, 2020). Previous studies have demonstrated that mitogenomes within the genus *Ophryotrocha* exhibit high dynamism in gene order (Tempestini *et al.*, 2020; Struck *et al.*, 2023). In this study, the gene order of *O. xiamenensis* was most similar to that of *O. japonica,* but differed from *O. labronica*, which indicated that gene arrangement differed between species (Table S3).

In the genus of *Ophryotrocha*, the phylogenetic tree based on all available genome of *Ophryotrocha* species benefited our understanding of their evolutionary process. Only the position of *O. diadema* changes between the two mitochondrial trees based on 13 PCGs or *cox1*/Histone3 sequences (Dahlgren *et al.*, 2001; Tempestini *et al.*, 2020; Pruitt, 2021). *O. xiamenensis*, *O. japonica* and *O. labronica*, showed the same gene order, and differed from other annelids, such as *O. adherens, O. diadema, O. robusta, Capitella teleta* (Tempestini *et al.*, 2020; Tilic and Rouse, 2024; Su *et al.*, 2025). Overall, our findings contribute to explore the evolution, the high biodiversity, and phylogenetic relationships among annelid taxa.

**Disclosure statement**

No potential conflict of interest was reported by the authors.

**Funding declaration**

This work was supported by Large Instruments Open Foundation of Minjiang University Project under MJUS2024K013.

**Author’s contribution**

Ruanni Chen and Jianming Chen designed the study. Ruanni Chen, Yuting Zhang, Ruoyu Liu, and Yiping Feng maintained the samples and wrote the manuscript. Yiping Feng, Wenting Lin and Fengqi Zhang conducted the experiments and performed the data analyses. Ruoyu Liu, Yuting Zhang, Jianming Chen, and Ruanni Chen reviewed the manuscript. All authors agreed to this final version of the manuscript.

**Ethical approval**

The material involved in the article does not involve ethical conflicts. This species is neither endangered on the CITES catalog nor collected from a natural reserve, so it did not need specific permissions or licenses. All collection and sequencing work were strictly executed under local legislation and related laboratory regulations to protect wild resources.

**Data availability statement**

The genome sequence data that support the findings of this study are openly available in GenBank of NCBI at https://www.ncbi.nlm.nih.gov/ under the accession no. PV831793. The chromatographic raw data used to generate the results are available at Zenodo: https://doi.org/10.5281/zenodo.17473716.

**References**

Alalykina, I. L., and N. E. Polyakova, 2022. New species of *Ophryotrocha* (Annelida: Dorvilleidae) associated with deep-sea reducing habitats in the Bering Sea, Northwest Pacific. Deep Sea Research Part II: Topical Studies in Oceanography 206**:** 105217.

Bernt, M., A. Donath, F. Jühling, F. Externbrink, C. Florentz *et al.*, 2013. MITOS: improved de novo metazoan mitochondrial genome annotation. Molecular phylogenetics and evolution 69**:** 313-319.

Chen, R., Y. Cheng, Y. Zhang and J. Chen, 2024. Identification and expression analysis of Oxfibrillin gene involved in the regeneration process of *Ophryotrocha xiamen* (Annelida, Dorcilleidae). Developmental & Comparative Immunology 151**:** 105102.

Chen, R., I. Mukhtar, S. Wei, S. Wu and J. Chen, 2022. Morphological and molecular features of early regeneration in the marine annelid *Ophryotrocha xiamen*. Scientific Reports 12**:** 1799.

Dahlgren, T. G., B. Akesson, C. Schander, K. M. Halanych and P. Sundberg, 2001. Molecular phylogeny of the model annelid *Ophryotrocha*. The Biological Bulletin 201**:** 193-203.

Grant, J. R., E. Enns, E. Marinier, A. Mandal, E. K. Herman *et al.*, 2023. Proksee: in-depth characterization and visualization of bacterial genomes. Nucleic acids research 51**:** W484-W492.

Pruitt, J., 2021 Phylogeny of *Ophryotrocha* (Annelida Dorvilleidae) Revisited, with Description of Six New Species from Eastern Pacific Seeps and Whalefalls. Master’s thesis, University of California San Diego.

Santovito, A., A. Pappalardo, A. Nota, M. Prearo and D. Schleicherová, 2023. Lymnaea stagnalis and *Ophryotrocha diadema* as model organisms for studying genotoxicological and physiological effects of benzophenone-3. Toxics 11**:** 827.

Su, X., D. Yang, X. Wu, Y. Sun, J.-W. Qiu *et al.*, 2025. Substantial mitochondrial gene order rearrangements and differential evolution rates within the family Capitellidae (Annelida). Zoosystematics and Evolution 101**:** 955-967.

Struck, T. H., A. Golombek, C. Hoesel, D. Dimitrov, A. H. Elgetany, 2023. Mitochondrial genome evolution in Annelida—a systematic study on conservative and variable gene orders and the factors influencing its evolution. Systematic Biology 72: 925-945.

Svensson, S. G. B., S. Meier, S. A. Mjøs, T. Strohmeier and H. M. Jansen, 2025. *Ophryotrocha craigsmithi* (Wiklund, Glower & Dahlgren, 2009) has a high capacity to modify lipids from aquaculture waste and synthesize long-chain polyunsaturated fatty acids. Aquaculture**:** 742746.

Tamura, K., D. Peterson, N. Peterson, G. Stecher, M. Nei *et al.*, 2011. MEGA5: molecular evolutionary genetics analysis using maximum likelihood, evolutionary distance, and maximum parsimony methods. Molecular biology and evolution 28**:** 2731-2739.

Tempestini, A., G. Massamba-N’Siala, F. Vermandele, N. Beaudreau, M. Mortz *et al.*, 2020. Extensive gene rearrangements in the mitogenomes of congeneric annelid species and insights on the evolutionary history of the genus *Ophryotrocha*. BMC genomics 21**:** 1-16.

Tilic, E., and G. W. Rouse, 2024. Hardly Venus’s servant-morphological adaptations of Veneriserva to an endoparasitic lifestyle and its phylogenetic position within Dorvilleidae (Annelida). Organisms Diversity & Evolution 24**:** 67-83.

Weigert A, Golombek A, Gerth M, Schwarz F, Struck TH, Bleidorn C., 2016. Evolution of mitochondrial gene order in Annelida. Mol Phylogenet Evol 94:196-206.

Won EJ, Rhee JS, Shin KH, Lee JS., 2013. Complete mitochondrial genome of the marine polychaete, *Perinereis nuntia* (Polychaeta, Nereididae). Mitochondrial DNA 24:342-3.

Zhang, D., F. Gao, I. Jakovlić, H. Zou, J. Zhang *et al.*, 2020. PhyloSuite: An integrated and scalable desktop platform for streamlined molecular sequence data management and evolutionary phylogenetics studies. Molecular ecology resources 20**:** 348-355.

Zhong, S., Zhao, Y., Zhang, Q., Chen, X., 2018. The complete mitochondrial genome of the cryptic species in peanut worm *Sipunculus nudus* (Sipuncula, Sipunculidae) from Beibu Bay. Mitochondrial DNA Part B 3(2): 484-485.
