# Supplementary material for: The complete mitochondrial genome of a marine polychaete, Ophryotrocha xiamenensis (Annelida: Dorvilleidae)
Source: Mitochondrial DNA B Resour. 2026 Mar 26;11(5):567–71. doi: 10.1080/23802359.2026.2647557 (PMC13022994; doi:10.1080/23802359.2026.2647557)
Supplement: Editing Certificate.pdf [file TMDN_A_2647557_SM7436.pdf]

Editing Certificate

This document certifies that the manuscript

The complete mitochondrial genome of a marine polychaete, Ophryotrocha xiamenensis (Annelida: Dorvilleidae)

prepared by the authors

Yiping Feng, Wenting Lin, Fengqi Zhang, Ruoyu Liu, Yuting Zhang, Jianming Chen, Ruanni Chen

was edited for proper English language, grammar, punctuation, spelling, and overall style by one or more of the highly qualified English speaking editors at AJE.

This certificate was issued on **March 10, 2026** and may be verified on the AJE website using the verification code **2054-AA01-7596-EC79-5B02** .

Neither the research content nor the authors' intentions were altered in any way during the editing process. Documents receiving this certification should be English-ready for publication; however, the author has the ability to accept or reject our suggestions and changes. To verify the final AJE edited version, please visit our verification page at aje.com/certificate. If you have any questions or concerns about this edited document, please contact AJE at support@aje.com.

AJE provides a range of editing, translation, and manuscript services for researchers and publishers around the world. For more information about our company, services, and partner discounts, please visit aje.com.
