# Supplementary material for: The complete mitochondrial genome of a marine polychaete, Ophryotrocha xiamenensis (Annelida: Dorvilleidae)
Source: Mitochondrial DNA B Resour. 2026 Mar 26;11(5):567–71. doi: 10.1080/23802359.2026.2647557 (PMC13022994; doi:10.1080/23802359.2026.2647557)
Supplement: Supplementary material.pdf [file TMDN_A_2647557_SM7435.pdf]

The complete mitochondrial genome of a marine polychaete, *Ophryotrocha xiamen* (Annelida: Dorvilleidae)

Yiping Feng[1, 2], Wenting Lin[2], Fengqi Zhang[2], Ruoyu Liu[1], Yuting Zhang[2], Jianming Chen*[2], Ruanni Chen*[2]

1 State Key Laboratory of Mariculture Breeding, Key Laboratory of Marine Biotechnology of Fujian Province, College of Marine Sciences, Fujian Agriculture and Forestry University, Fuzhou 350002, China
2 Fujian Key Laboratory on Conservation and Sustainable Utilization of Marine Biodiversity, Fuzhou Institute of Oceanography, College of Geography and Oceanography, Minjiang University, Fuzhou, 350108, China

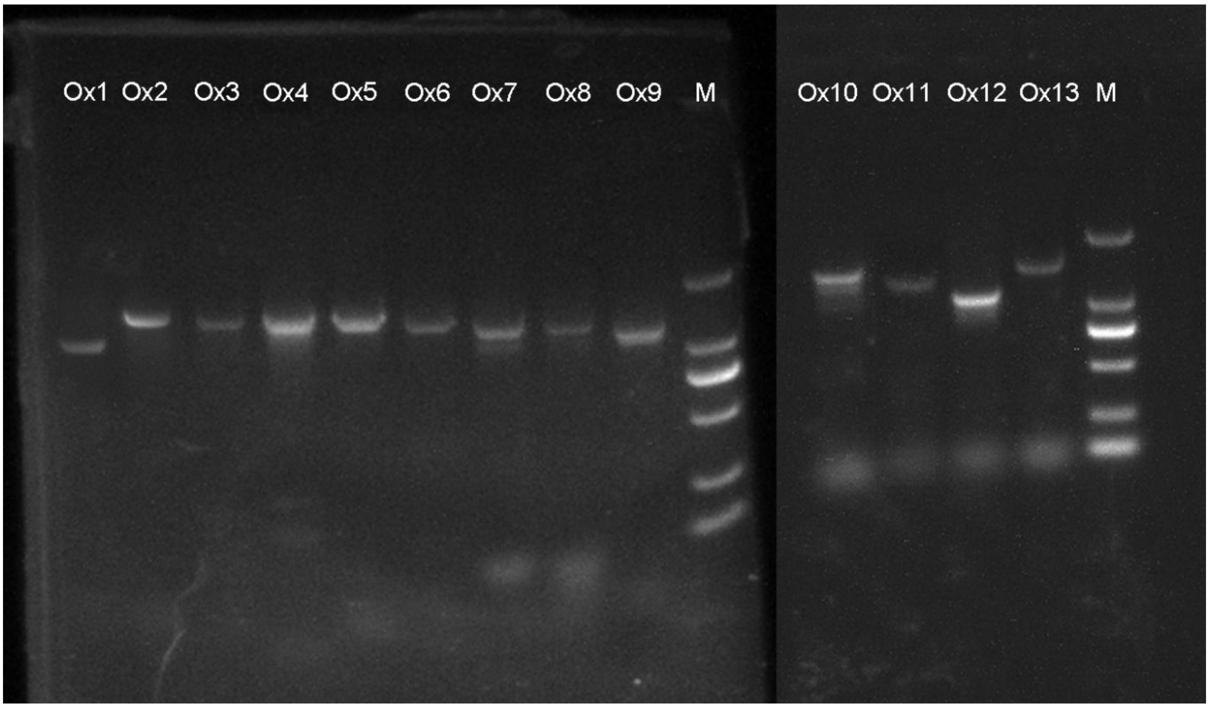

Figsure S1. The electrophoresis image of eight PCR fragments of Ophryotrocha xiamen mitochondrial genome in 1.2% agarose gel. Ox1-10 stands for primer 1 to primer 10. M stands for marker (DL2000).

Figsure S2. Structural analysis of mitochondrial genes in *Ophryotrocha xiamen* by using TBtools-II software.

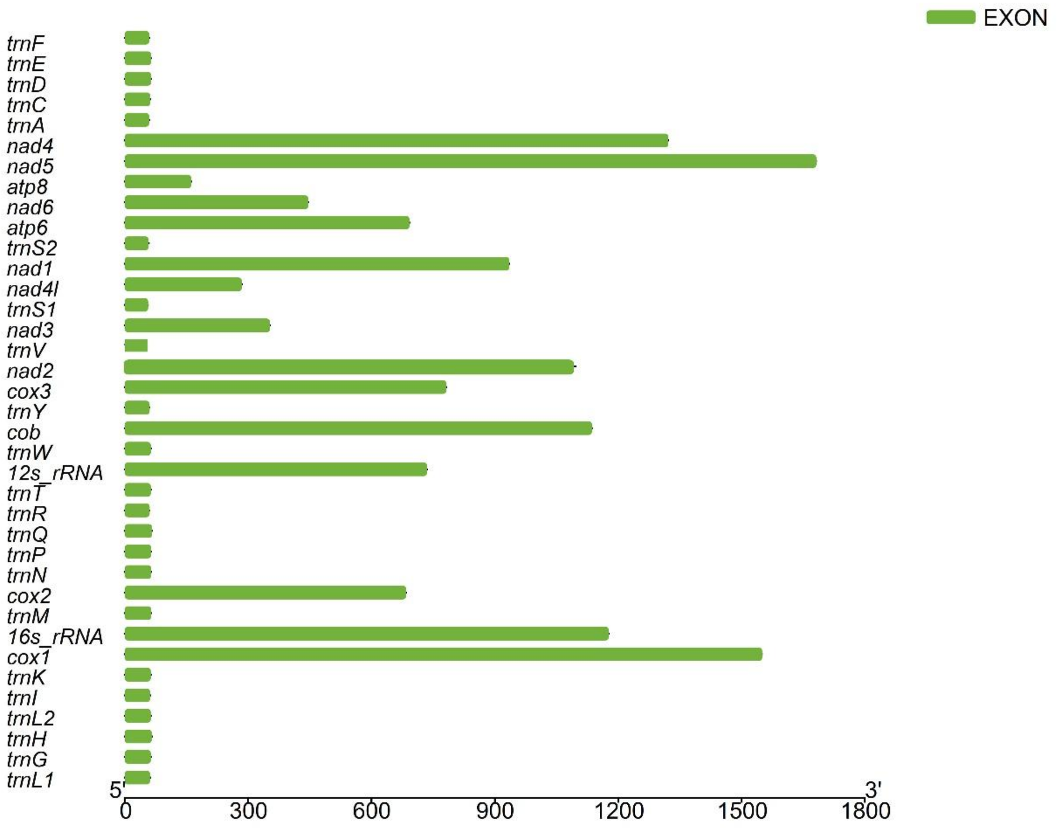

Table S1. Estimates of Evolutionary Divergence between Sequences.

	O. adherens	*O. diadema*	*O. japonica*	*O. labronica*	*O. puerilis*	*O. robusta*	*O. xiamenensis*	*P. dumerilii*	*O. fusiformis*	*C. variopedatus*	*S. nudus*
O. adherens		0.009	0.010	0.008	0.008	0.007	0.010	0.013	0.011	0.010	0.011
O. diadema	0.559		0.010	0.011	0.009	0.008	0.010	0.012	0.013	0.011	0.010
O. japonica	0.630	0.687		0.005	0.013	0.006	0.002	0.011	0.009	0.010	0.013
O. labronica	0.615	0.688	0.349		0.010	0.008	0.005	0.010	0.009	0.010	0.012
O. puerilis	0.552	0.619	0.699	0.691		0.009	0.012	0.010	0.011	0.010	0.011
O. robusta	0.581	0.655	0.484	0.502	0.641		0.006	0.011	0.008	0.009	0.014
O. xiamenensis	0.623	0.684	0.058	0.349	0.685	0.477		0.011	0.008	0.011	0.015
P. dumerilii	0.638	0.650	0.740	0.695	0.638	0.691	0.740		0.010	0.011	0.012

O. fusiformis	0.631	0.687	0.624	0.607	0.677	0.589	0.617	0.629		0.012	0.011
C. variopedatus	0.642	0.675	0.749	0.747	0.663	0.694	0.746	0.616	0.654		0.012
S. nudus	0.673	0.660	0.765	0.788	0.671	0.745	0.767	0.573	0.664	0.614	

The number of base substitutions per site from between sequences are shown. Standard error estimates are shown above the diagonal. Analyses were conducted using the K2p model.

Table S2. Genome annotation for *Ophryotrocha xiamen*.

Ophryotrocha xiamen					
**Name**	**Start**	**Stop**	**Strand**	**Length**	**Codons**
cox1	1	1527	+	1527	ATT/TAA
trnC(gca)	1538	1600	+	63	
trnN(gtt)	1601	1665	+	65	
nad4l	1682	1966	+	285	ATG/TAA
nad4	1960	3282	+	1323	ATG/TAA
trnS2(tga)	3288	3346	+	59	
trnA(tgc)	3347	3406	+	60	
trnM(cat)	3405	3469	+	65	
12s rRNA	3467	4202	+	736	
trnG(tcc)	4206	4269	+	64	
trnV(tac)	4272	4326	+	55	
16s rRNA	4327	5403	+	1077	
trnY(gta)	5404	5463	+	60	
trnL1(tag)	5479	5540	+	62	
nad1	5541	6452	+	912	ATG/TAA
trnK(ttt)	6455	6518	+	64	
trnI(gat)	6517	6578	+	62	
nad3	6579	6926	+	348	ATG/TAG
trnE(ttc)	7019	7983	+	65	
trnP(tgg)	7087	7151	+	65	
trnL2(taa)	7176	7239	+	64	
trnD(gtc)	7523	7587	+	65	
ncr	7588	8987	+	1399	
trnF(gaa)	8988	9044	+	57	
trnT(tgt)	9051	9114	+	64	

cox2	9154	9838	+	685	ATG/CGT
trnW(tca)	9863	9926	+	64	
atp8	9930	10091	+	162	ATG/TAG
cox3	10092	10874	+	783	ATG/TAA
trnQ(ttg)	10877	10943	+	67	
nad6	10944	11390	+	447	ATG/TAA
cytb	11394	12530	+	1137	GTG/TAG
atp6	12544	13236	+	693	ATG/TAG
trnR(tcg)	13252	13312	+	61	
trnH(gtg)	13314	13379	+	66	
nad5	13380	15062	+	1683	ATG/TAA
trnS1(tct)	15062	15120	+	59	
nad2	15122	16078	+	958	ATG/TAG

ncr=non-coding region

Table S3. Comparison of gene orders between *O. xiamenensis*, *O. japonica* and *O. labronica*.

	O. xiamenensis	***O. japonica***	***O. labronica***
1	*cox1*	*cox1*	*cox1*
2	*trnC(gca)*	*trnC(gca)*	*trnC(gca)*
3	*trnN(gtt)*	*trnN(gtt)*	*trnN(gtt)*
4	*nad4l*	*nad4l*	*nad4l*
5	*nad4*	*nad4*	*nad4*
6	*trnS2(tga)*	*trnS2(tga)*	*trnS2(tga)*
7	*trnA(tgc)*	*trnA(tgc)*	*trnA(tgc)*
8	*trnM(cat)*	*trnM(cat)*	*trnM(cat)*
9	*12s rRNA*	*12s rRNA*	*12s rRNA*
10	*trnG(tcc)*	*trnG(tcc)*	*trnG(tcc)*
11	*trnV(tac)*	*trnV(tac)*	*trnV(tac)*
12	*16s rRNA*	*16s rRNA*	*trnP(tgg)* ?
13	*trnY(gta)*	*trnY(gta)*	*16s rRNA*
14	*trnL1(tag)*	*trnL1(tag)*	*trnY(gta)*
15	*nad1*	*nad1*	*trnL1(tag)*
16	*trnK(ttt)*	*trnK(ttt)*	*trnL2(taa)*
17	*trnI(gat)*	*trnI(gat)*	*nad1*
18	*nad3*	*nad3*	*trnK(ttt)*
19	*trnE(ttc)*	*trnE(ttc)*	*trnI(gat)*

20	*trnP(tgg)*	*trnP(tgg)*	*nad3*
21	*trnL2(taa)*	*trnL2(taa)*	*trnE(ttc)*
22	*trnD(gtc)*	-	*trnD(gtc)*
23	*ncr*	*ncr*	*ncr*
24	*trnF(gaa)*	*trnF(gaa)*	*trnF(gaa)*
25	*trnT(tgt)*	*trnT(tgt)*	*trnT(tgt)*
26	*cox2*	*cox2*	*cox2*
27	*trnW(tca)*	*trnW(tca)*	*trnW(tca)*
28	*atp8*	*atp8*	*atp8*
29	*cox3*	*cox3*	*cox3*
30	*trnQ(ttg)*	*trnQ(ttg)*	*trnQ(ttg)*
31	*nad6*	*nad6*	*nad6*
32	*cytb*	*cytb*	*cytb*
33	*atp6*	*atp6*	*atp6*
34	*trnR(tcg)*	*trnR(tcg)*	*trnR(tcg)*
35	*trnH(gtg)*	*trnH(gtg)*	*trnH(gtg)*
36	*nad5*	*nad5*	*nad5*
37	*trnS1(tct)*	*trnS1(tct)*	*trnS1(tct)*
38	*nad2*	*nad2*	*nad2*
