# Supplementary material for: The complete mitochondrial genome of a marine polychaete, Ophryotrocha xiamenensis (Annelida: Dorvilleidae)
Source: Mitochondrial DNA B Resour. 2026 Mar 26;11(5):567–71. doi: 10.1080/23802359.2026.2647557 (PMC13022994; doi:10.1080/23802359.2026.2647557)
Supplement: clean copy The complete mitochondrial genome of a marine polychaete.docx [file TMDN_A_2647557_SM7434.docx]

**The complete mitochondrial genome of a marine polychaete, *Ophryotrocha xiamenensis* (Annelida: Dorvilleidae)**

Yiping Feng^1, 2^, Wenting Lin^2^, Fengqi Zhang^2^, Ruoyu Liu^1^, Yuting Zhang^2^, Jianming Chen*^2^, Ruanni Chen*^2^

1 State Key Laboratory of Mariculture Breeding, Key Laboratory of Marine Biotechnology of Fujian Province, College of Marine Sciences, Fujian Agriculture and Forestry University, Fuzhou 350002, China

2 Fujian Key Laboratory on Conservation and Sustainable Utilization of Marine Biodiversity, Fuzhou Institute of Oceanography, College of Geography and Oceanography, Minjiang University, Fuzhou, 350108, China

**Correspondence:**

(1) Ruanni Chen, chenruanni@163.com; (2) Jianming Chen, chenjm@mju.edu.cn.

**Abstract**

*Ophryotrocha xiamenensis*, a small opportunistic worm, is an appropriate model for investigating regeneration and evolution. In this study, the complete mitochondrial genome of *O. xiamenensis* was sequenced using the Sanger platform. It is 16,111 bp in length and consists of 13 protein-coding genes (PCGs), 22 transfer RNA genes, 2 ribosomal RNA genes and a noncoding region. Phylogenetic analysis based on concatenated sequences of all 13 PCGs using the maximum-likelihood and Bayesian methods, placed *O. xiamenensis* within the ‘labronica’ clade and the order of gene arrangement was the same as that of *O. japonica*. This study contributes to the development of genetic resources and advances the understanding of phylogenetic resolution within the genus *Ophryotrocha*.

**Keywords**

Mitogenome, *Ophryotrocha* *xiamenensis*, phylogenetic analysis, Bayesian

**1. Introduction**

*Ophryotrocha xiamenensis* Chen, 2022, belongs to the order Eunicida, family Dorvilleidae, genus *Ophryotrocha*, and is a newly found species (Chen et al., 2022). The genus *Ophryotrocha* Claparède & Mecznikow, 1869 has been found in a wide range of habitats from shallow water to the deep sea (Alalykina and Polyakova, 2022; Svensson et al., 2025). Owing to their ability to survive laboratory conditions, high fecundity, short generation times, and rapid individual growth rates, some species of *Ophryotrocha* have been used as model organisms among marine invertebrates, in the fields of genetics, reproduction, development, and regeneration (Tempestini et al., 2020; Santovito et al., 2023; Chen et al., 2024). Molecular-based taxonomic approaches have been used to identify morphologically similar species in the genus *Ophryotrocha*. Approximately 97 species in this genus, excluding *O. xiamenensis*, have been described according to GBIF data (https://www.gbif.org/); however, the complete mitochondrial genomes of only 6 species have been sequenced (Tempestini et al., 2020). The complete mitochondrial genome may serve as a foundation for thorough evolutionary investigations. The study of the genomic sequences of intermediate forms may shed light on the nuances of molecular evolution mechanisms as well as the conditions of relic species survival. In this study, we explored the complete mitochondrial genome of *O. xiamenensis* and examined its phylogenetic relationships with other species.

**2. Materials and methods**

**2.1 Sample collections**

Specimen samples (Figure 1) were collected from Baicheng Bay, Xiamen, China (118.08E, 24.44 N), and cultured in our laboratory for more than five years. The species was identified by pairwise comparisons of *cox1* and *histone H3* sequences (Chen et al., 2022). All the samples are now deposited in the Fujian Key Laboratory on Conservation and Sustainable Utilization of Marine Biodiversity, College of Geography and Oceanography, Minjiang University under voucher number F3-7 (contact Ruanni Chen, chenruanni@mju.edu.cn).


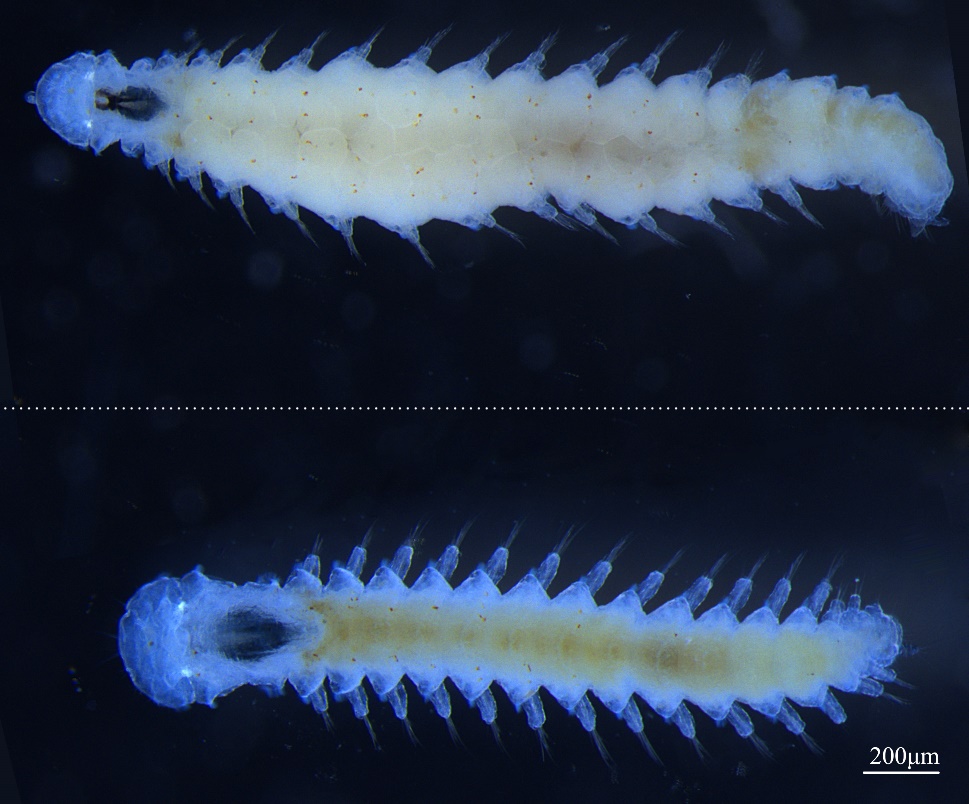


Figure 1. Photographs of live *O. xiamenensis*, smaller male and larger female, in dorsal view (Ruanni Chen, Minjiang University).

**2.2 Sequencing and annotation**

Genomic DNA was extracted from whole worms using a TIANamp Marine Animals DNA Kit (TIANGEN, Beijing, China). Universal and specific PCR primers were designed on the basis of the *cox1* sequence from *O. xiamenensis* and conserved sequences from the genus *Ophryotrocha* using MEGA5.0 (Table 1). The fragments were amplified using Premix Taq (RR901, TaKaRa Co., Dalian, China) with initial denaturation at 95 °C for 4 min, followed by 35 cycles at 95 °C for 30 s, annealing at 45–58 °C for 1 min, and extension at 72 °C for 1 min, with a final elongation at 70 °C for 10 min after the last cycle. The amplified fragments were separated through 1.2% agarose gel electrophoresis and sequenced using the Sanger sequencing method (Figure S1, supplementary material). For assembly, the annotation of the mitogenome was performed using MITOS2 and EMBOSS Transeq (Bernt et al., 2013). Exact positions of protein-coding genes (PCGs) and rRNAs were found by searching for ORFs (employing genetic code 2, the invertebrate mitochondrion) or by homologous comparison. The circular mitochondrial genome of *O. xiamenensis* was then visualized using the Proksee web server (Grant et al., 2023).

Table 1. The primers used in sequencing mitochondrial genomes of *Ophryotrocha xiamenensis*.

| No. | Primer | Sequence 5’-3’ | Tm (℃) | Lengths |
| --- | --- | --- | --- | --- |
| 1 | Ox1 | CGTTTTGAAGTGGCGRRGATGT  TWCCCCTTAAWGAMCCTAATAA | 58 | 1155 |
| 2 | Ox2 | TAAATGTTRTCACGKAATCCKTTYC | 55 | 1541 |
|  |  | CAACCATAATTAATATCMCGAGA |  |  |
| 3 | Ox3 | CAATTATTAAGGGGGTTGTTCCTC | 53 | 1598 |
|  |  | CAAATCAATTCACATAACTCCTTG |  |  |
| 4 | Ox4 | ATAACTGCAGGGCATATTGT | 58 | 1673 |
|  |  | TAAYWGGGTGGGGGTAGGGAAAAAG |  |  |
| 5 | Ox5 | GKGTATAGRTAYCGRATAAT | 45 | 1581 |
|  |  | CCAATATCTTTATGATTWGT |  |  |
| 6 | Ox6 | AACKTSRKWTTTTTWRTTTTTA | 48 | 1454 |
|  |  | GTAAAMACATCMGGRTAATCT |  |  |
| 8 | Ox7 | ATGAGCGGTGATCCCGTGTTCAG | 50 | 1504 |
|  |  | GTTTCACTAGTATACTTAAAACTAG |  |  |
| 7 | Ox8 | CTATAGTAAGCTCCTTACCTTTG | 55 | 1346 |
|  |  | CCAATGTGGATTGTCAAATTAT |  |  |
| 9 | Ox9 | TTAGCAGTTTTAGGGGAGTAATCT | 53 | 1258 |
|  |  | TTCTAGTCCCCAATTAAGGAAC |  |  |
| 10 | Ox10 | CTGCCCGGTGCTTTTTATAGT | 54 | 1167 |
|  |  | TGATATTTATCCTATGCCAAAACA |  |  |
| 11 | Ox11 | CTCTAAGTATGCGCTTTTAGGG | 55 | 1142 |
|  |  | TCTTAACCACGAAAAAGTCACG |  |  |
| 12 | Ox12 | TCGTTTAAGAGCCTTCTATAGTT | 51 | 870 |
|  |  | ATTCGGTATAAGGTACTCACTCA |  |  |
| 13 | Ox13 | GGCATTAATTATAACAACACTATG | 49 | 1393 |
|  |  | GGAAAACCGTTAATTGTAGTATAG |  |  |

**2.3 Phylogenetic analysis**

Molecular phylogenetic analyses were performed with datasets from all 13 PCGs through MEGA 5.05 software (Tamura et al., 2011). Each PCG was separately aligned, after which the concatenated and poorly aligned regions were removed. In total, 11 terminal taxa were used in the analyses—7 species from the genus *Ophryotrocha*, including *O. xiamenensis*, and 3 species from the other families of Polychaeta; the tree was rooted using *Sipunculus nudu*. The aligned sequences were used as datasets to generate the genetic distance using Kimura’s two-parameter (K2P) model. On the basis of the K2P distances (Table S1), we calculated the interspecific genetic differences among the closest taxa. The phylogenetic trees were constructed by the maximum likelihood method (ML) using MEGA software, with 1,000 bootstrap pseudo replicates. ModelFinder was used to select the best-fit model for Bayesian analysis by using PhyloSuite version 1.2.3(Zhang et al., 2020). Bayesian inference (BI) phylogenies were inferred under the TVM+I+G+F model (2 parallel runs, 200000 generations), in which the initial 25% of the sampled data were discarded as burn-in. FigTree v1.4.4 was used to visualize the tree. There are neither trans-splicing nor cis-splicing genes in the mitochondrial genome.

**3. Results**

We assembled a complete mitochondrial genome of *O. xiamenensis* which has been maintained in our laboratory for more than five years. The complete mitogenome (GenBank Accession No. PV831793) is 16,111 bp long and A + T biased. Sanger sequencing chromatograms of the *O. xiamenensis* mitogenome are provided in the supplementary material. Thirty-seven genes were identified from the mitogenome sequence, including 13 PCGs, 2 ribosomal genes (12S and 16S), 22 transport RNA genes, and 1 noncoding region (NCR). The NCR of the mitochondrial genome was located between *trnD* and *trnF*. The total base composition was A (26.8%), T (37.8%), G (24.1%), and C (11.3%). All the genes were coded on the plus strand (Figure 2) and no introns were found (see Figure S2, supplementary material). The total length of the 13 PCGs and NCR was 10,994 bp and 6 overlapping regions were present in the whole mitochondrial genome, including 1 to 17 bp (see Table S2, supplementary material). Most of the genes started with an ATG codon and ended with a TAA/TAG stop codon. Specifically, for *cox1* and *cytb,* ATT and GTG as start codons.


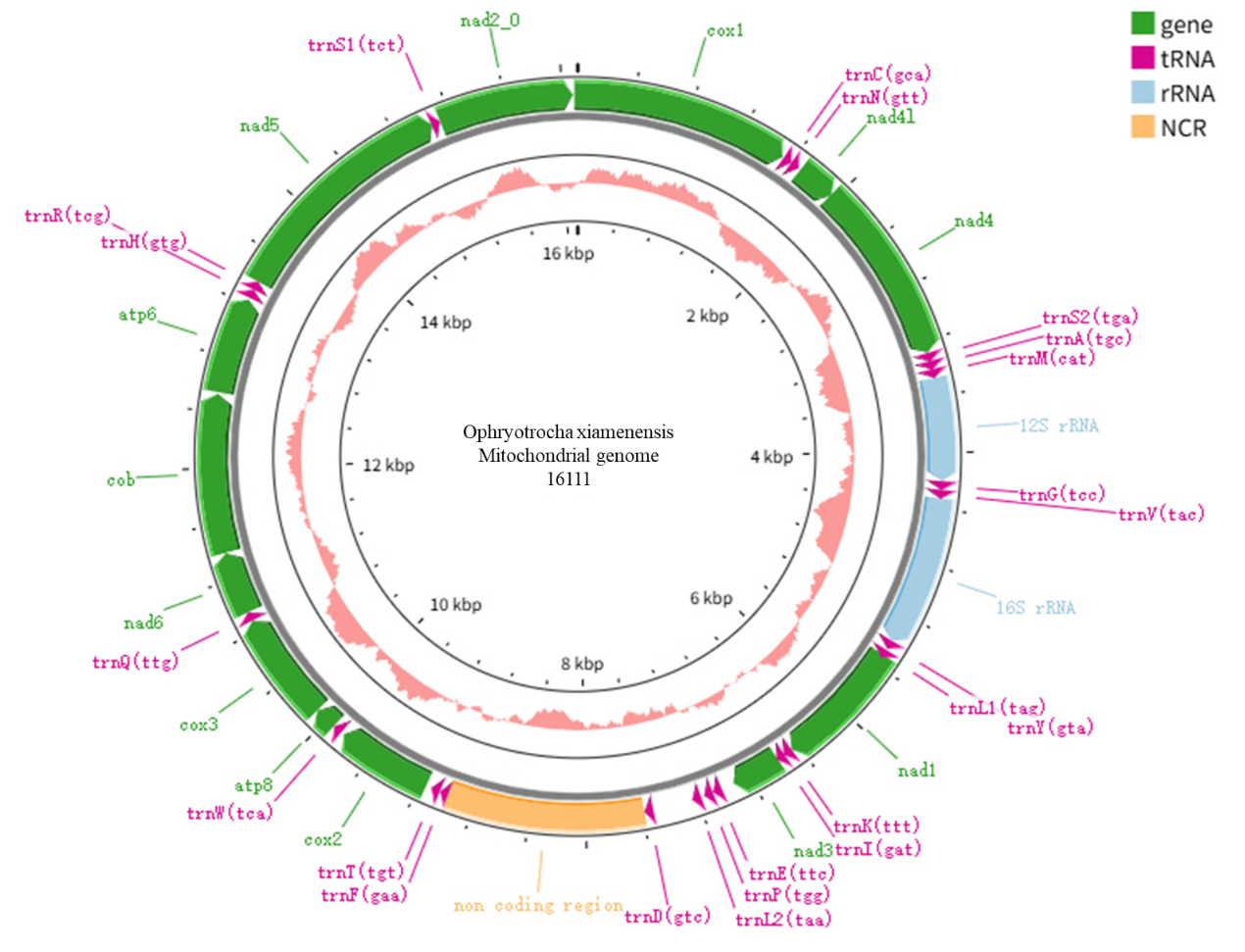


Figure 2. Circular map of the complete mitochondrial genome of *Ophryotrocha xiamenensis*. The complete mitochondrial genome was 16,111 bp in length. Genes were shown with standard abbreviations. The outer circle indicated the plus strand (outer line) and the minus strand (inner line), while all genes were coded on plus strand. The inner pink bars indicated the GC content, and the middle line represented 50%.

After the poorly aligned positions were removed, a total of 12550 bp of 13 PCGs were used for phylogenetic analyses. Phylogenetic analyses revealed similar tree topologies regardless of whether the ML or Bayesian approach was used; therefore, only the results from the Bayesian analysis are shown (Figure 3). Maximum-likelihood and BI revealed phylogenetic relationships within the genus *Ophryotrocha*, indicating that *O. xiamenensis* fell within the ‘labronica’ clade. *O. xiamenensis* presented the same gene order as *O. japonica* did, except for *trnD*. The 13 PCGs of *O. xiamenensis* and *O. japonica* were in the same order. The positions of *trnL2*, *trnD*, and *trnP* could help to identify related species in the ‘labronica’ clade. The mitochondrial genome, especially its gene arrangement, is valuable for studying evolutionary relationships among the genus *Ophryotrocha*.


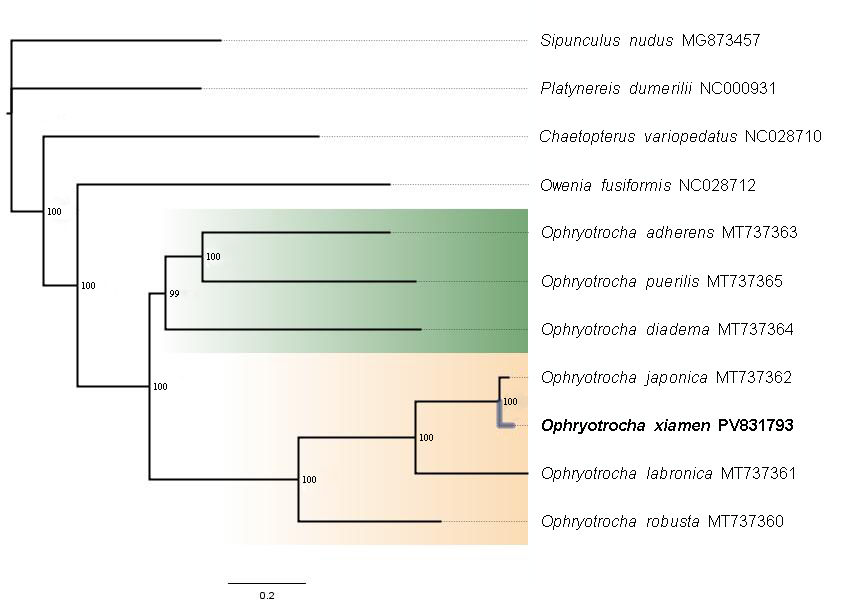


Figure 3. Phylogenetic tree was constructed using 13 protein-coding genes of the complete mitochondrial genome through PhyloSuite v1.2.3. Bayesian posterior probabilities were shown at each node. The following sequences were used: *Sipunculus nudus* MG873457 (Zhong et al., 2018), *Platynereis dumerilii* NC000931 (Won et al., 2013), *Chaetopterus variopedatus* NC028710 (Weigert et al.,2015), *Owenia fusiformis* NC028712 (Weigert et al.,2015), *Ophryotrocha adherens* MT737363 (Tempestini et al.,2020), *Ophryotrocha puerilis* MT737365 (Tempestini et al.,2020), *Ophryotrocha diadema* MT737364 (Tempestini et al.,2020), *Ophryotrocha japonica* MT737362 (Tempestini et al.,2020), *Ophryotrocha xiamenensis* PV831793, *Ophryotrocha labronica* MT737361(Tempestini et al.,2020), and *Ophryotrocha robusta* MT737360 (Tempestini et al.,2020).

**4. Discussion and Conclusion**

The genus *Ophryotrocha* has been used as a model organism in several fields of comparative and evolutionary biology of marine invertebrates. Herein, the complete mitogenome of *O. xiamenensis* was sequenced and found to be 16,111 bp in length, including 13 PCGs, 22 tRNAs, 2 rRNAs and 1 NCR. Notably, *cox1* and *cytb* used ATT and GTG were used as start codons for *cox1* and *cytb*, which were also found in *O. japonica* and *O. labornica* (Tempestini et al., 2020). Previous studies have demonstrated that mitogenomes within the genus *Ophryotrocha* exhibit high dynamism in terms of gene order (Tempestini et al., 2020; Struck et al., 2023). In this study, the gene order of *O. xiamenensis* was most similar to that of *O. japonica* but differed from that of *O. labronica*, indicating that gene arrangement varies between species (Table S3).

The phylogenetic tree based on all available genomes of species within the genus *Ophryotrocha* has improved our understanding of their evolutionary process. Only the position of *O. diadema* changes between the two mitochondrial trees on the basis of 13 PCGs or *cox1*/Histone H3 sequences (Dahlgren et al., 2001; Tempestini et al., 2020; Pruitt, 2021). *O. xiamenensis*, *O. japonica* and *O. labronica*, have the same gene order, and differ from other annelids, such as *O. adherens, O. diadema, O. robusta,* and *Capitella teleta* (Tempestini et al., 2020; Tilic and Rouse, 2024; Su et al., 2025). Overall, our findings contribute to the exploration of the evolution, high biodiversity, and phylogenetic relationships among annelid taxa.

**Disclosure statement**

No potential conflict of interest was reported by the authors.

**Funding declaration**

This work was supported by Large Instruments Open Foundation of Minjiang University Project under MJUS2024K013.

**Author’s contribution**

Ruanni Chen and Jianming Chen designed the study. Ruanni Chen, Yuting Zhang, Ruoyu Liu, and Yiping Feng maintained the samples and wrote the manuscript. Yiping Feng, Wenting Lin and Fengqi Zhang conducted the experiments and performed the data analyses. Ruoyu Liu, Yuting Zhang, Jianming Chen, and Ruanni Chen reviewed the manuscript. All authors agreed to this final version of the manuscript.

**Ethical approval**

The material involved in the article does not involve ethical conflicts. This species is neither endangered on the CITES catalog nor collected from a natural reserve, so it did not need specific permissions or licenses. All collection and sequencing work were strictly executed under local legislation and related laboratory regulations to protect wild resources.

**Data availability statement**

The genome sequence data that support the findings of this study are openly available in GenBank of NCBI at https://www.ncbi.nlm.nih.gov/ under the accession no. PV831793. The chromatographic raw data used to generate the results are available at Zenodo: https://doi.org/10.5281/zenodo.17473716.

**References**

Alalykina, I. L., and N. E. Polyakova, 2022. New species of *Ophryotrocha* (Annelida: Dorvilleidae) associated with deep-sea reducing habitats in the Bering Sea, Northwest Pacific. Deep Sea Research Part II: Topical Studies in Oceanography 206**:** 105217.

Bernt, M., A. Donath, F. Jühling, F. Externbrink, C. Florentz et al., 2013. MITOS: improved de novo metazoan mitochondrial genome annotation. Molecular phylogenetics and evolution 69**:** 313-319.

Chen, R., Y. Cheng, Y. Zhang and J. Chen, 2024. Identification and expression analysis of Oxfibrillin gene involved in the regeneration process of *Ophryotrocha xiamen* (Annelida, Dorcilleidae). Developmental & Comparative Immunology 151**:** 105102.

Chen, R., I. Mukhtar, S. Wei, S. Wu and J. Chen, 2022. Morphological and molecular features of early regeneration in the marine annelid *Ophryotrocha xiamen*. Scientific Reports 12**:** 1799.

Dahlgren, T. G., B. Akesson, C. Schander, K. M. Halanych and P. Sundberg, 2001. Molecular phylogeny of the model annelid *Ophryotrocha*. The Biological Bulletin 201**:** 193-203.

Grant, J. R., E. Enns, E. Marinier, A. Mandal, E. K. Herman et al., 2023. Proksee: in-depth characterization and visualization of bacterial genomes. Nucleic acids research 51**:** W484-W492.

Pruitt, J., 2021 Phylogeny of *Ophryotrocha* (Annelida Dorvilleidae) Revisited, with Description of Six New Species from Eastern Pacific Seeps and Whalefalls. Master’s thesis, University of California San Diego.

Santovito, A., A. Pappalardo, A. Nota, M. Prearo and D. Schleicherová, 2023. Lymnaea stagnalis and *Ophryotrocha diadema* as model organisms for studying genotoxicological and physiological effects of benzophenone-3. Toxics 11**:** 827.

Su, X., D. Yang, X. Wu, Y. Sun, J.-W. Qiu et al., 2025. Substantial mitochondrial gene order rearrangements and differential evolution rates within the family Capitellidae (Annelida). Zoosystematics and Evolution 101**:** 955-967.

Struck, T. H., A. Golombek, C. Hoesel, D. Dimitrov, A. H. Elgetany, 2023. Mitochondrial genome evolution in Annelida—a systematic study on conservative and variable gene orders and the factors influencing its evolution. Systematic Biology 72: 925-945.

Svensson, S. G. B., S. Meier, S. A. Mjøs, T. Strohmeier and H. M. Jansen, 2025. *Ophryotrocha craigsmithi* (Wiklund, Glower & Dahlgren, 2009) has a high capacity to modify lipids from aquaculture waste and synthesize long-chain polyunsaturated fatty acids. Aquaculture**:** 742746.

Tamura, K., D. Peterson, N. Peterson, G. Stecher, M. Nei et al., 2011. MEGA5: molecular evolutionary genetics analysis using maximum likelihood, evolutionary distance, and maximum parsimony methods. Molecular biology and evolution 28**:** 2731-2739.

Tempestini, A., G. Massamba-N’Siala, F. Vermandele, N. Beaudreau, M. Mortz et al., 2020. Extensive gene rearrangements in the mitogenomes of congeneric annelid species and insights on the evolutionary history of the genus *Ophryotrocha*. BMC genomics 21**:** 1-16.

Tilic, E., and G. W. Rouse, 2024. Hardly Venus’s servant-morphological adaptations of Veneriserva to an endoparasitic lifestyle and its phylogenetic position within Dorvilleidae (Annelida). Organisms Diversity & Evolution 24**:** 67-83.

Weigert A, Golombek A, Gerth M, Schwarz F, Struck TH, Bleidorn C., 2016. Evolution of mitochondrial gene order in Annelida. Mol Phylogenet Evol 94:196-206.

Won EJ, Rhee JS, Shin KH, Lee JS., 2013. Complete mitochondrial genome of the marine polychaete, *Perinereis nuntia* (Polychaeta, Nereididae). Mitochondrial DNA 24:342-3.

Zhang, D., F. Gao, I. Jakovlić, H. Zou, J. Zhang et al., 2020. PhyloSuite: An integrated and scalable desktop platform for streamlined molecular sequence data management and evolutionary phylogenetics studies. Molecular ecology resources 20**:** 348-355.

Zhong, S., Zhao, Y., Zhang, Q., Chen, X., 2018. The complete mitochondrial genome of the cryptic species in peanut worm *Sipunculus nudus* (Sipuncula, Sipunculidae) from Beibu Bay. Mitochondrial DNA Part B 3(2): 484-485.
